# Expression of a Novel P22 ORFan Gene Reveals the Phage Carrier State in *Salmonella* Typhimurium

**DOI:** 10.1371/journal.pgen.1003269

**Published:** 2013-02-14

**Authors:** William Cenens, Mehari T. Mebrhatu, Angella Makumi, Pieter-Jan Ceyssens, Rob Lavigne, Rob Van Houdt, François Taddei, Abram Aertsen

**Affiliations:** 1 Laboratory of Food Microbiology, Department of Microbial and Molecular Systems (M2S), Faculty of Bioscience Engineering, Katholieke Universiteit Leuven, Leuven, Belgium; 2 Laboratory of Gene Technology, Department of Biosystems, Faculty of Bioscience Engineering, Katholieke Universiteit Leuven, Leuven, Belgium; 3 Unit of Microbiology, Belgian Nuclear Research Centre (SCK•CEN), Mol, Belgium; 4 INSERM, U1001, Université Rene Descartes, Paris, France; Universidad de Sevilla, Spain

## Abstract

We discovered a novel interaction between phage P22 and its host *Salmonella* Typhimurium LT2 that is characterized by a phage mediated and targeted derepression of the host *dgo* operon. Upon further investigation, this interaction was found to be instigated by an ORFan gene (designated *pid* for phage P22 encoded instigator of *dgo* expression) located on a previously unannotated moron locus in the late region of the P22 genome, and encoding an 86 amino acid protein of 9.3 kDa. Surprisingly, the Pid/*dgo* interaction was not observed during strict lytic or lysogenic proliferation of P22, and expression of *pid* was instead found to arise in cells that upon infection stably maintained an unintegrated phage chromosome that segregated asymmetrically upon subsequent cell divisions. Interestingly, among the emerging siblings, the feature of *pid* expression remained tightly linked to the cell inheriting this phage carrier state and became quenched in the other. As such, this study is the first to reveal molecular and genetic markers authenticating pseudolysogenic development, thereby exposing a novel mechanism, timing, and populational distribution in the realm of phage–host interactions.

## Author Summary

Viruses of bacteria, also referred to as (bacterio)phages, are the most abundant biological entity on earth and have a tremendous impact on the ecology of their hosts. It has traditionally been recognized that upon infection by a temperate phage the host cell is forced either to produce and release new virions during lytic development or to replicate and segregate the phage chromosome together with its own genetic material during lysogenic development. These developmental paths are orchestrated by a dedicated set of phage–host interactions that are able to sense and redirect host cell physiology. In addition to this classical bifurcation of temperate phage development, many studies on phage biology in natural ecosystems hypothesize the existence and significance of stable phage carrier cells that are not engaged in either lytic or lysogenic proliferation. Using *Salmonella* Typhimurium and phage P22 as a model system, we provide substantial evidence authenticating the existence of the phage carrier state and demonstrate that this state (i) is asymmetrically inherited among carrier cell siblings and (ii) enables the execution of a novel phage–host interaction that is not encountered during lytic or lysogenic proliferation.

## Introduction

Due to billions of years of co-evolution and their overpowering abundance in the biosphere, viruses of bacteria (i.e. bacteriophages or phages) have a profound impact on the conduct and ecology of their hosts [1], [2]. Lytic proliferation of phages for example can affect host mutation rates [3], structure microbial consortia [4], and contribute significantly to the global biogeochemical carbon flux [5]. Lysogenic proliferation as stable prophages, on the other hand, increases the genetic repertoire and genome plasticity of the host, thereby often extending its adaptive potential in terms of virulence and ecological fitness [6].

While the basic molecular events and genetic circuitry behind lytic and lysogenic development have traditionally received a lot of attention and are reasonably well understood for a number of model phages [7]–[9], the increasing wealth of novel phage genes with no known homologs and function nevertheless suggests an unforeseen intricacy in phage – host interactions [1], [10]. Furthermore, in many ecological niches phage – host associations often appear to defy the classical bifurcation into strict lytic or lysogenic development, as a large number of reports indicate a lysogeny-independent but stable co-existence between phages and their hosts. These phenomena are often vaguely referred to as pseudolysogeny, and hypothesize the existence of stable “phage carrier” cells in which the incoming phage has temporarily refrained from lytic or lysogenic development [11]. This suspended state is believed to play an important role in the long term survival strategy of viruses, as it might (i) prevent poor replication or even degradation of the phage chromosome in a host that is too starved to support further steps in lytic or lysogenic development, and/or (ii) provide a transient intracellular refuge for the phage chromosome in environments characterized by low host densities and short capsid half-lives [12], [13]. Despite its ecological importance [11], [14], however, no formal molecular evidence currently exists for the presence of such a state, let alone its possible impact on the physiology of the cell.

In this study, we extend on the intricacy of phage – host interactions and provide both genetic and direct cell biological evidence for the existence of a dedicated pseudolysogenic state in the *Salmonella* Typhimurium – phage P22 model system.

## Results

### Mu*d*K mutagenesis of *Salmonella* Typhimurium LT2 reveals a clone that responds to infection by phage P22

**Figure 1:**
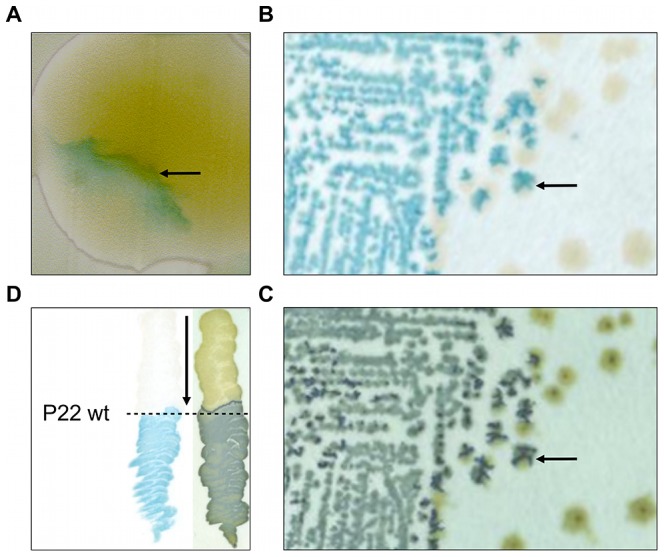
Initial observation of an LT2 clone (designated LT2K7) carrying a *lacZ* translational fusion that responds to P22 infection. In clockwise orientation: (A) Photograph of a P22 *HT105/1 int201* infected colony of LT2K7, showing inhomogeneous distribution of LacZ activity as visualized by the blue color that results from hydrolysis of X-Gal on LB X-Gal agar (cfr. arrow). (B) Typical streak out on LB X-Gal agar of a colony shown in panel A. (C) An exact replica on green indicator agar of the colonies shown in panel B. The arrow in B and C indicates the position of the same colony, indicating LacZ activity (shown in panel B) to overlap with phage infection (shown in panel C). (D) Streaking of LT2K7 (following the direction of the arrow) across a suspension of wild-type P22 (P22 wt; indicated by a dashed line) on LB X-Gal agar to reveal LacZ activity (left panel), and green indicator agar to reveal phage infection (right panel).

During routine screening of a Mu*d*K based *lacZ* promoter-trap library in *Salmonella* Typhimurium LT2 on LB X-Gal agar plates, our attention was drawn to a colony displaying an inhomogeneous distribution of LacZ activity (i.e. blue coloration; Figure 1A) that was neither symmetrical, nor sectorial. Moreover, after streaking out on new LB X-Gal plates, this particular clone segregated both into plain white colonies and colonies with an irregular blue coloration similar to that of the parent colony (Figure 1B). Interestingly, however, when the latter colonies were replica-plated on green indicator agar, the blue patches on LB X-Gal agar overlapped perfectly with the dark green sites of cell lysis that were revealed by the green indicator agar (compare Figure 1B and 1C). As we reasonably assumed this cell lysis to stem from infection by residual P22 *HT105/1 int201* transducing phage that was initially used to deliver the Mu*d*K element during construction of the library, we hypothesized LacZ activity of the isolated clone to be triggered by exposure to phage P22.

In order to further examine this phenotype, the Mu*d*K insertion of the corresponding clone was transduced into a fresh LT2 strain and a phage-free transductant (designated LT2K7) was streaked across wild-type P22 (P22 wt) on LB X-Gal agar (Figure 1D). As a result, we found LT2K7 to turn from white to blue upon encountering P22, suggesting that phage P22 is causally involved in triggering *lacZ* expression in LT2K7.

### Mu*d*K of LT2K7 maps to the *dgoT* gene

**Figure 2:**
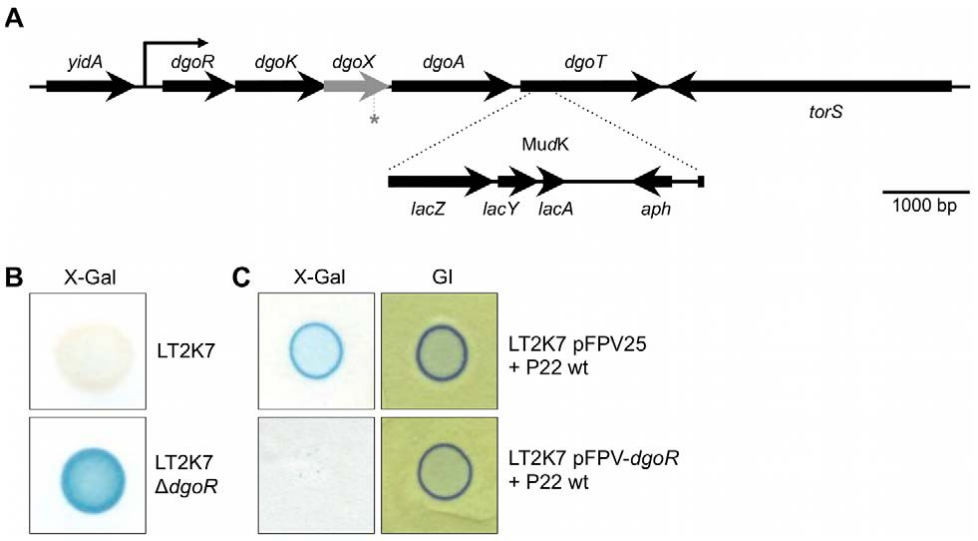
The *dgoRKAT* operon and its derepression in LT2. (A) Scheme showing the position of the Mu*d*K element (not drawn to scale) generating a translational *lacZ* reporter fusion to the *dgoT* gene in strain LT2K7 (i.e. *dgoT*::Mu*d*K). Please note that the grey arrow corresponds to an open reading frame compromised by a −1 frame shift at the position marked with an asterisk (*). (B) Deletion of the *dgoR* gene in LT2K7 yields constitutive expression of the *dgoT*::Mu*d*K fusion, as shown by the difference in LacZ activity (i.e. blue color) between a spot of LT2K7 and its Δ*dgoR* derivative grown on LB X-Gal agar. (C) Overexpression of *dgoR* interferes with activation of the *dgoT*::Mu*d*K fusion by P22 infection, as a plaque of P22 wt grown on a lawn of LT2K7 pFPV-*dgoR* fails to display LacZ activity (i.e. blue color) on LB X-Gal agar (left panel). A plaque of P22 wt grown on LT2K7 pFPV25 (i.e. empty vector) was included as a control. A similar experiment performed on green indicator agar (GI; right panel) confirms the actual infection of both strains by P22 wt.

The Mu*d*K insertion site of LT2K7 was mapped to the *dgoRKAT* operon. DNA sequence analysis revealed that the Mu*d*K insertion resulted in a translational fusion of the *lacZ* reporter gene to *dgoT* (Figure 2A). The *dgoR* gene located at the beginning of the operon is predicted to function as an autorepressor [15] and indeed LT2K7 Δ*dgoR* constitutively expressed the *dgoT*::Mu*d*K fusion (Figure 2B), regardless of infection by P22 wt. Furthermore, increasing the level of DgoR by providing the corresponding gene on a multicopy plasmid (pFPV-*dgoR*) was able to abolish induction of *dgoT*::Mu*d*K by P22 wt, but had no obvious effect on phage infection *per se* (Figure 2C). These data suggest that infection by P22 interferes with autorepression of the *dgo* operon in LT2.

### A P22 moron locus is responsible for specifically triggering *dgo* expression in LT2

**Figure 3:**
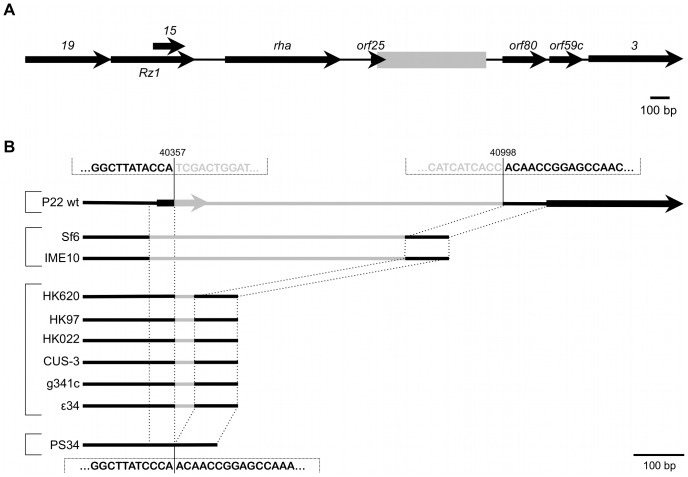
Genomic context of the P22 *pid* locus. (A) Position of the region containing the *pid* locus (boxed in gray), situated between *orf25* and *orf80* in the late region of the P22 wt genome. Expression of *pid* proceeds in the direction opposite to that of its surrounding genes. (B) Comparison of this specific locus, situated between nucleotide 40357 and 40998 of the P22 genome (NCBI Reference Sequence: NC_002371.2), throughout related phages. Black lines indicate regions of homology while grey lines are only homologous within phages grouped by brackets.

Subsequently, we noticed that derepression of *dgoT*::Mu*d*K upon phage infection was a feature supported by P22, but not by another *S. enterica* specific temperate phage such as ES18 (Figure S1). This raised the possibility that induction of the *dgo* operon stemmed from a genetic circuit in P22, rather than from a generic host response to phage infection. To examine this, a plasmid library of random P22 genomic fragments was screened for loci able to render the LT2K7 indicator strain blue on LB X-Gal. As such, a 521 bp P22 fragment could eventually be obtained that triggered *dgoT*::Mu*d*K upon conditional expression (using the arabinose inducible P*_BAD_* promoter) in LT2K7. More specifically, this fragment was found to correspond to a small and unannotated locus situated between *orf25* and *orf80* in the late region of the P22 genome [16], [17] (Figure 3A, boxed region), and was subsequently designated as *pid* (for phage P22 encoded instigator of *dgo* expression).

Interestingly, close inspection of the *pid* region revealed it to be a genuine moron locus [6], as it is integrated at a site where related phages have either no (cfr. PS34 in Figure 3B) or another insert (Figure 3B). In addition, the *pid* locus is further characterized (i) by the fact that it is divergently transcribed relative to its surrounding genes, indicating that its regulatory control might deviate from that of the late region, and (ii) by a 3′ Rho-independent transcriptional termination site.

### The P22 *pid* locus encodes a small ORFan protein that triggers expression of the LT2 *dgo* operon

**Figure 4:**
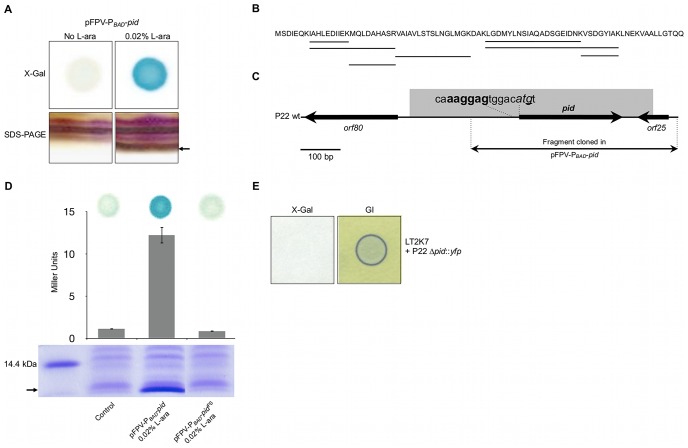
Identification of the P22 Pid protein. (A) Conditional expression with 0.02% L-arabinose (L-ara) of the *pid* locus from pFPV-P*_BAD_-pid* yields (i) LacZ activity (i.e. blue color) caused by induction of the *dgoT*::Mu*d*K fusion in a spot of LT2K7 pFPV-P*_BAD_-pid* grown on LB X-Gal agar (upper right panel) as opposed to a non-induced control (upper left panel), and (ii) the appearance of a small protein (its position is marked by the arrow) on a silver stained SDS-PAGE of total protein extract of LT2 pFPV-P*_BAD_-pid* (lower right panel) as opposed to a non-induced control (lower left panel). (B) Protein sequence of Pid, in which peptide signatures identified by mass-spectrometry are underlined. (C) Location of the *pid* gene on the P22 genome, with indication of its predicted start codon (shown in italic) and Shine-Dalgarno sequence (shown in bold). The nucleotide that was deleted to introduce a −1 frame-shift (yielding pFPV-P*_BAD_-pid* ^FS^) is underlined. (D) Plasmids pFPV-P*_BAD_-pid* and pFPV-P*_BAD_-pid* ^FS^ were compared with respect to their ability to yield (i) Pid protein in LT2, as determined by SDS-PAGE of total protein extracts (lower panel; position of Pid is marked by the arrow), and (ii) LacZ activity caused by induction of the *dgoT*::Mu*d*K fusion in LT2K7, as determined qualitatively by blue coloration on LB X-Gal agar (upper panel) and quantitatively by Miller Units (middel panel; means ± standard error of three independent experiments are shown). For comparison, an uninduced pFPV-P*_BAD_-pid* plasmid was used as a control. (E) Deletion of the *pid* gene in P22 (i.e. P22 Δ*pid*::*yfp*) abolishes induction of the *dgoT*::Mu*d*K fusion upon infection of LT2K7, as indicated by absence of LacZ activity (i.e. blue color) in the corresponding plaque on LB X-Gal agar (left panel), but does not interfere with phage infection, as indicated by green indicator agar (GI; right panel).

During our efforts to discriminate whether the *pid* locus encoded a small regulatory RNA or a small protein, we discovered the appearance of a distinct low molecular weight protein band on SDS-PAGE upon triggering transcription of the locus from a plasmid (pFPV-P*_BAD_*-*pid*) (Figure 4A). Moreover, sequencing of this protein indeed revealed peptide signatures encoded by one of the possible reading frames of the moron locus (Figure 4B). While the stop codon of this open reading frame could be inferred, the start codon was predicted by the presence of an upstream canonical Shine-Dalgarno sequence (AAGGAG) [18] (Figure 4C). Importantly, introduction of a −1 frame shift in the start codon (Figure 4C) simultaneously abolished both expression of the characteristic protein band and induction of *dgoT*::Mu*d*K in LT2K7 (Figure 4D), establishing this 86 amino acid and 9.23 kDa protein (termed Pid; Figure 4B) (and not a small RNA species putatively originating from the same locus) as the actual trigger of the interaction. It should be noted that subsequent deletion of the *pid* open reading frame in P22 correspondingly abolished induction of the *dgo* operon upon infection (Figure 4E), but had no noticeable impact on the ability to develop lytically or lysogenically.

### The Pid/*dgo* interaction is not supported during strict lytic or lysogenic propagation of P22

**Figure 5:**
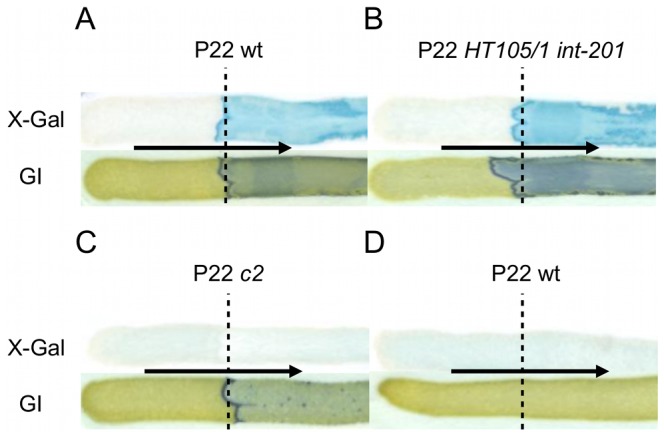
The Pid/*dgo* interaction is not supported during strict lytic or lysogenic development of P22. LT2K7 (A, B, C) and a P22 wt lysogen of LT2K7 (D) was streaked (in the direction of the arrow) across a suspension (indicated by a dashed line) of P22 wt (A, D), P22 *HT105/1 int-201* (B) and P22 *c2* (C) on either LB X-Gal agar (upper panels) in order to visualize LacZ activity caused by induction of the *dgoT*::Mu*d*K fusion, or green indicator agar (lower panels) in order to visualize phage infection.

Since upon infection the propagation of P22 wt can either proceed lytically or lysogenically, we wondered which of these two distinct developmental routes would actually mount the Pid/*dgo* interaction (Figure 5A) in the cell. Surprisingly, however, *dgoT*::Mu*d*K expression was completely absent both when LT2K7 was subjected to obligate lytic infection with P22 *c2* (Figure 5C) or when the reporter strain carried P22 wt as a prophage (Figure 5D). The latter finding is in fact consistent with our initial observation of the Pid/*dgo* interaction being fully supported by the P22 *HT105/1 int-201* transducing phage (Figure 1A, 1B and Figure 5B) despite its inability to integrate in the host chromosome as a prophage.

**Figure 6:**
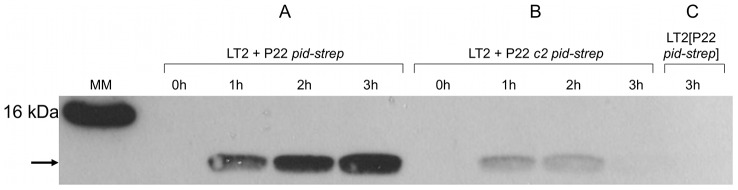
Expression of Pid protein during P22 infection. Western blot analysis of strep-tagged Pid produced during the indicated time after infecting exponential phase cultures of LT2 at MOI = 0.1 with (A) P22 *pid-strep* or (B) P22 *c2 pid-strep*, or (C) in early exponential phase cultures of LT2 carrying P22 *pid-strep* as a prophage. The position of Pid is indicated by a black arrow, while the molecular marker (MM) shows the position of the 16 kDa reference.

To further corroborate this finding, we extended the P22 *pid* open reading frame with a strep-tag encoding sequence (leading to P22 *pid-strep*) to facilitate Pid detection by western blot, and checked whether the observed absence of *dgoT*::Mu*d*K expression also correlated with attenuated levels of Pid. In agreement with the results above (Figure 5), Pid production was abundant in LT2 infected with P22 *pid-strep* (Figure 6A), while it was severely attenuated in LT2 infected with the obligate lytic P22 *c2 pid-strep* derivative (Figure 6B) and completely absent in LT2 carrying P22 *pid-strep* as a prophage (Figure 6C).

**Figure 7:**
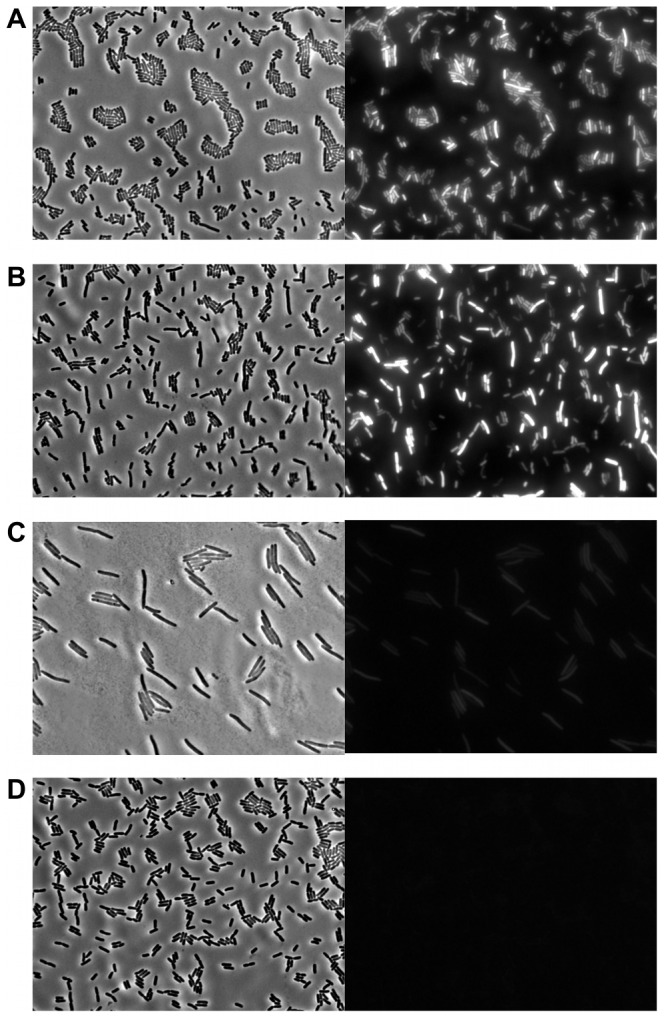
Evidence for *pid* transcription during P22 infection at the single cell level. Phase contrast (left panels) and corresponding YFP epifluorescence (right panels) micrographs of exponential phase cultures of LT2 either (A) 3 h after infection with P22 Δ*pid*::*yfp*, (B) 3 h after infection with P22 Δ*int* Δ*pid*::*yfp*, (C) 2 h after infection with P22 *c2* Δ*pid*::*yfp* (just before general cell lysis occurred), or (D) carrying P22 Δ*pid*::*yfp* as a prophage. Infections with P22 (A, B and C) were performed at MOI = 0.1.

To determine whether or not compromised Pid production stemmed from attenuated *pid* transcription, the *pid* open reading frame of P22 was replaced with the *yfp* fluorescent reporter gene, and the resulting phage (i.e. P22 Δ*pid*::*yfp*, carrying *yfp* under the control of the native *pid* promoter) was used to interact with LT2. In agreement with our previous findings (Figure 5 and Figure 6), cells infected with an obligate lytic derivative of P22 Δ*pid*::*yfp* (i.e. P22 *c2* Δ*pid*::*yfp*) only displayed very faint fluorescence in the few minutes before cell lysis (Figure 7C), while cells carrying P22 Δ*pid*::*yfp* as a prophage displayed no detectable fluorescence (Figure 7D). On the contrary, cells infected with P22 Δ*pid*::*yfp* (Figure 7A) or its *int* derivative (i.e. P22 Δ*int* Δ*pid*::*yfp*) (Figure 7B) clearly showed a plethora of cells exhibiting YFP expression to different extents.

Interestingly, the finding that expression of *pid* and subsequent derepression of the *dgo* operon are not supported during lytic or lysogenic propagation of P22 strongly suggests that the Pid/*dgo* interaction might be dedicated to a different state of P22 development.

### *pid* expression is tightly linked with cells in the phage carrier state

**Figure 8:**
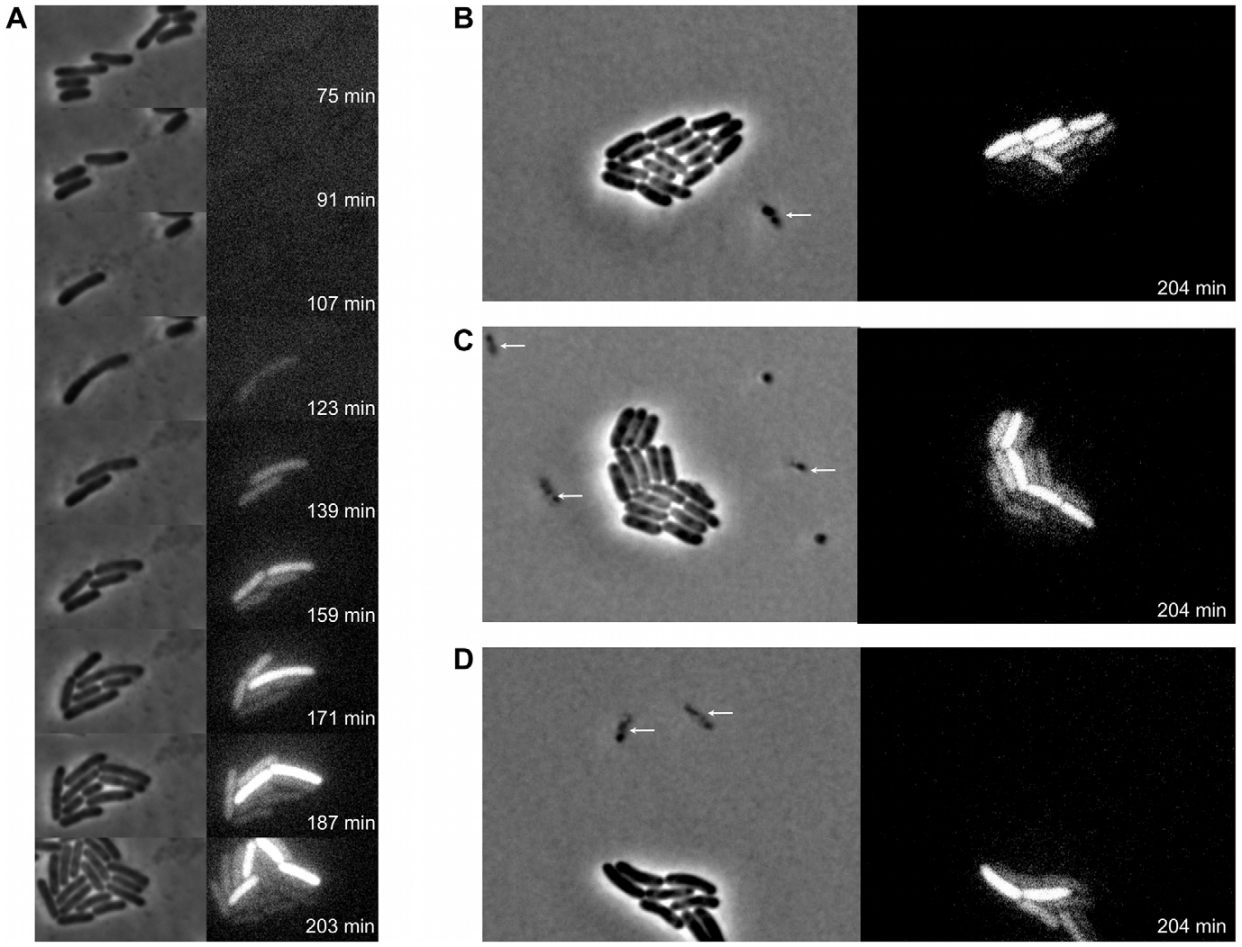
Expression of P22 *pid* segregates asymmetrically between siblings. Exponential phase cultures of LT2 were infected with P22 Δ*pid*::*yfp* (MOI = 0.1) and chased after 30 minutes with the virulent P22 H5 mutant (MOI = 20) to lyse cells not destined for non-lytic development of P22 Δ*pid*::*yfp*. (A) Time-lapse fluorescence microscopy image sequence of an LT2 cell destined for non-lytic development of P22 Δ*pid*::*yfp*. (B–D) Images from clonal microcolonies similar to the one emerging from a single cell in panel A, and also displaying asymmetrical segregation of *yfp* expression. Please note that the surrounding cells (indicated by arrows) are lysing due to lytic infection with either P22 Δ*pid*::*yfp* or P22 H5. Phase contrast (left panels) and corresponding YFP epifluorescence (right panels) images are shown, and the time after infection with P22 Δ*pid*::*yfp* is indicated on the frames.

Spurred by the above observations, time-lapse fluorescence microscopy was used to more closely examine the timing and dynamics of *pid* expression during infection of LT2 with P22 Δ*pid*::*yfp* at single cell resolution. While this approach demonstrated that the *pid* locus indeed became expressed in lineages emerging from non-lytic infection with the reporter phage, it also revealed that this expression was a feature that subsequently segregated asymmetrically between siblings (Figure 8). Surprisingly, in fact, only one individual within the growing lineage consistently displayed the ability to express *pid*, thereby revealing an unprecedented timing and populational distribution of this phage – host interaction. It should also be noted that disruption of the *int* gene in P22 Δ*pid*::*yfp* did not affect the timing nor the asymmetric distribution of *pid* expression (Figure S2), corroborating that the actual chromosomal integration event leading to the establishment of a prophage was not required for this phenomenon.

**Figure 9:**
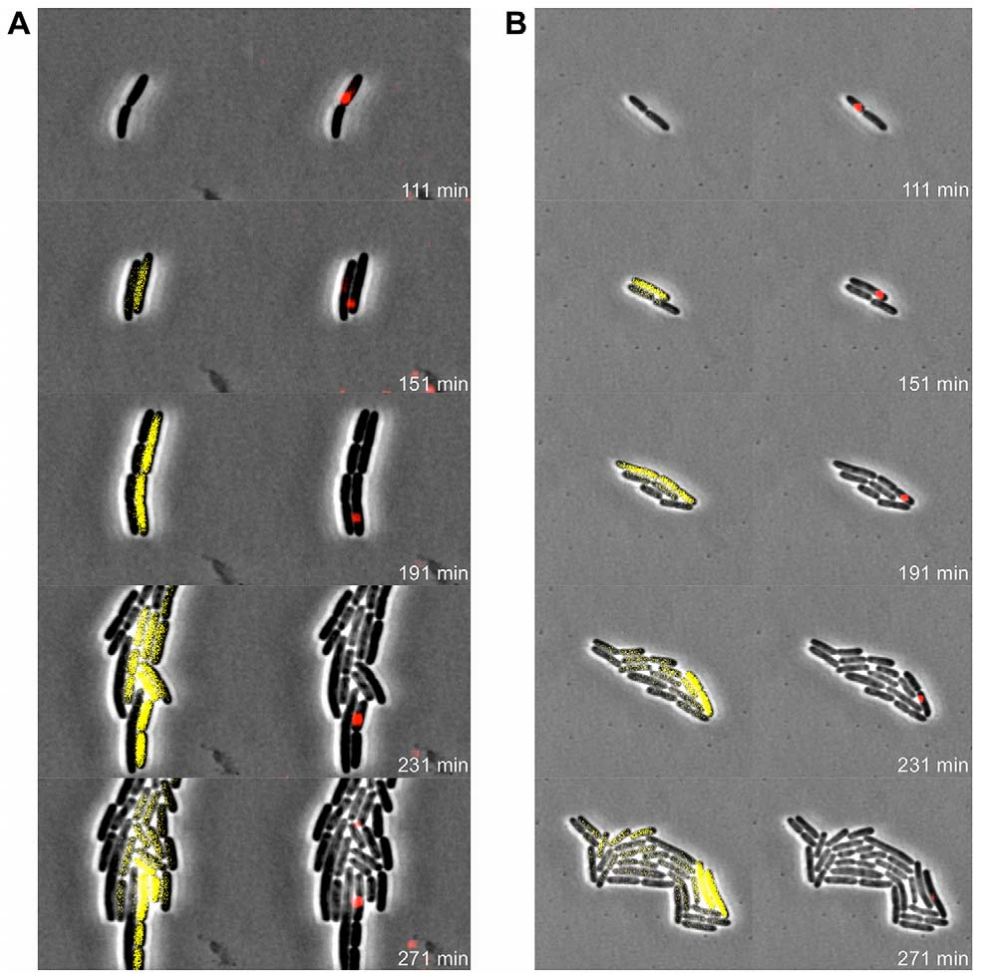
Pid expression is linked to phage carrier cells. Exponential phase cultures of LT2 pCW-*mCherry*-*parB* were infected with P22 Δ*pid*::*yfp parS* (MOI = 0.1) and chased after 30 minutes with the virulent P22 H5 mutant (MOI = 20) to lyse cells not destined for non-lytic development of P22 Δ*pid*::*yfp parS*. Two independent time-lapse fluorescence microscopy image sequences (A and B) of an LT2 pCW-*mCherry*-*parB* cell destined for non-lytic development of P22 Δ*pid*::*yfp parS* are shown. The corresponding phase contrast images are either superimposed with YFP epifluorescence images visualizing *pid* expression (left panels) or mCherry epifluorescence images visualizing the presence of the P22 chromosome(s) (right panels), and the time after infection is indicated on the frames. Please note that only the carrier cell produces new YFP, while non-carrier segregants passively acquire preformed YFP by cytoplasmic diffusion, and further dilute it upon subsequent cell divisions.

In order to more closely examine the possible role of the P22 chromosome in this peculiar asymmetric segregation phenotype, P22 Δ*pid*::*yfp* was equipped with a *parS* site (resulting in P22 Δ*pid*::*yfp parS*), allowing its whereabouts during infection to become fluorescently tractable in an LT2 strain expressing the ParB protein fused to mCherry (i.e. LT2 pCW-*mCherry-parB*). Interestingly, soon after infection of LT2 pCW-*mCherry-parB* with P22 Δ*pid*::*yfp parS*, a single and coherent mCherry cloud appeared in cells destined for non-lytic infection (Figure 9A and 9B), indicative for the presence of one (or possibly more) P22 chromosome(s). Furthermore, upon subsequent cell divisions, this cloud became asymmetrically segregated between siblings, with *pid* expression remaining tightly linked to the cell inheriting and carrying the unintegrated P22 chromosome(s) (Figure 9). The gradual dilution of YFP molecules in siblings not inheriting this phage carrier state is consistent with the heterogeneity in YFP fluorescence in liquid cultures of LT2 infected with P22 Δ*pid*::*yfp* observed earlier (cfr. Figure 7).

## Discussion

Given the penetration and importance of bacteriophages in global ecology, understanding their possible associations with a host is of tremendous importance. In this report, the *S.* Typhimurium – phage P22 model system yielded both molecular and genetic evidence authenticating the existence of a dedicated phage carrier state in which an unintegrated phage chromosome is stably maintained in the cell and asymmetrically inherited by only one of the siblings upon further divisions. This behavior differs fundamentally from cells undergoing lytic or lysogenic phage development, which are forced either to lyse after the production of new virions or to symmetrically segregate the prophage chromosome (integrated in the host chromosome or existing as a stable episome) among siblings [19], [20], respectively.

The phage carrier (or pseudolysogenic) state is believed to have a tremendous impact on phage ecology, as the ability to postpone the commitment to lytic or lysogenic development might improve phage survival in inhospitable environments [11]–[14]. Specifically with regard to the biology of phage P22, our findings at the single cell level are in remarkable agreement with very early observations made by Zinder, who anticipated that upon infection P22 could be maintained in a pseudolysogenic form during several generations before integrating itself as a prophage [21]. Despite the long-standing assumption of its alleged existence and its ecological importance, however, the phage carrier state has so far hardly been documented from a molecular or genetic point of view. In fact, although it has been proposed that the phage remains idle or inert while being in this state [12], [13], our results on the contrary provide the first evidence that a dedicated phage – host interaction (as exemplified by Pid/*dgo*) can be mounted in phage carrier cells. Clearly, the existence of dedicated genetic programs that are executed solely in phage carrier cells substantiates their biological significance and allows them to differentiate from uninfected cells or cells destined for lytic or lysogenic development.

On itself, the induction of the LT2 *dgo* operon by the P22 Pid ORFan protein is also peculiar, since only a very limited number of phage – host interactions have so far been discovered in which the phage deliberately and specifically interferes with host gene expression. Indeed, in currently recognized interactions, phage encoded functions either (i) hijack cellular machinery and generally shut down host gene expression to support phage reproduction during lytic proliferation [22], or (ii) contribute virulence factors that support the pathogenicity of the host during lysogenic development [6], [23]. A notable exception was only recently described for λ lysogens of *E. coli*, in which the λ CI repressor was shown to compromise cellular gluconeogenesis by physically obstructing the host *pckA* promoter [24].

Interestingly, the *dgo* operon encodes proteins involved in the uptake and metabolism of D-galactonate, which is considered to be an important source of carbon and energy during intracellular survival and proliferation of *Salmonella spp.* [25]. Moreover, a *dgoT* knock-out was correspondingly found to attenuate the virulence of *S. enterica* serovar Choleraesuis in pigs [26]. It remains to be established, however, how exactly the Pid/*dgo* interaction is mounted within the carrier state, and whether it would endow carrier cells with increased virulence or rather constitutes a way for the phage to decide on how long to maintain this state.

In summary, our results authenticate the existence of the phage carrier state as a distinct developmental route in phage biology that differs from strict lytic or lysogenic propagation. The phenotypic consequences of the interactions taking place in phage carrier cells are likely to provide the missing link in the proper and accurate interpretation of phage – host dynamics occurring throughout microbial ecosystems.

## Materials and Methods

### Strains and growth conditions

Bacterial strains, phages and plasmids used throughout this study are listed in Table 1. For culturing bacteria, Lysogeny Broth (LB; [27]) medium was used either as a broth or as agar plates after the addition of 15% (for spreading plates) or 7% (for soft-agar plates) agar. Cultures were grown in LB broth for 15–20 h at 37°C under well-aerated conditions (200 rpm on a rotary shaker) to reach stationary phase. Exponential phase cultures were in turn prepared by diluting stationary phase cultures 1/100 or 1/1000 in fresh pre-warmed broth, and allowing further incubation at 37°C. When appropriate, the following chemicals (Applichem, Darmstadt, Germany) were added to the growth medium at the indicated final concentrations: ampicillin (100 µg/ml; Ap^100^), chloramphenicol (30 µg/ml; Cm^30^), kanamycin (50 µg/ml; Km^50^), tetracycline (20 µg/ml; Tc^20^), glucose (0.02%), L-arabinose (0.02%), and 5-bromo-4-chloro-3-indolyl-β-D-galactopyranoside (X-Gal; 40 µg/ml).

**Table 1:** Strains, phages, and plasmids used in this study.

Name	Characteristic	Source or reference
**Strains**		
DH5α	*E. coli* F- φ80*lacZ*ΔM15 Δ(*lacZYA*-*argF*) U169*endA*1*recA*1*hsdR*17*deoRthi*-1*supE*441^−^ *gyrA*96*relA*1	Laboratory collection
LT2	*Salmonella* Typhimurium LT2 wild-type	[46]
LT2K7	LT2 *dgoT::*mu*d*K	This study
LT2 Δ*dgoR*	LT2 Δ*dgoR*	This study
LT2K7 Δ*dgoR*	LT2 *dgoT::mudK*Δ*dgoR*	This study
LT2[P22]	LT2 P22 lysogen	This study
LT2[P22 Δ*pid*::*yfp*]	LT2 P22 Δ*pid*::*yfp* lysogen	This study
LT2[P22 *pid-strep*]	LT2 P22 *pid-strep* lysogen	This study
**Phages**		
P22 wt	Wild-type P22 phage	SGSC^a^
P22 *c2*	Clear mutant of P22 affected in C2 repressor	SGSC^a^
P22 H5	Virulent derivative of P22	Kelly Hughes (University of Utah, USA)
P22 *HT105/1 int-201*	Integration deficient mutant of P22 used for generalized transduction	Kelly Hughes (University of Utah, USA)
P22 Δ*pid::yfp*	*pid* replaced by *yfp*	This study
P22 Δ*int* Δ*pid*::*yfp*	*pid* replaced by *yfp*, *int* deletion	This study
P22 *c2* Δ*pid*::*yfp*	*pid* replaced by *yfp*, truncated C2 repressor	This study
P22 *pid-strep*	C-terminal fusion of *strep-*tag to *pid*	This study
P22 *c2 pid-strep*	C-terminal fusion of *strep*-tag to *pid*, truncated C2 repressor	This study
P22 Δ*pid*-*yfp parS*-*cat*	*pid* replaced by *yfp*, *parS-cat* inserted between *gtrC* and *9*	This study
ES18		[47]
**Plasmids**		
pFPV25	Encodes promoterless *gfp*	[48]
pFPV-P*_BAD_*-*gfp*	Encodes GFP under control of an arabinose-inducible promoter	[31]
pFPV-P*_BAD_* -*pid*	Encodes Pid under control of an arabinose-inducible promoter	This study
pFPV-P*_BAD_-pid^FS^*	As in pFPV-P*_BAD_-pid* but harboring a frame shift in the start codon of *pid*	This study
pFPV-*dgoR*	Encodes DgoR under its native promoter	This study
pAc	*yfp-frt-cat-frt* template for recombineering of *yfp*	[37]
pCP20	Encodes Flp for recombining *frt* sites	[36]
pKD46	Encodes Lambda red genes under control of arabinose inducible promoter	[35]
pKD3	Harbors *frt-cat-frt* site for construction of deletions by recombineering	[35]
pALA2705	Encodes GFP-ParB under control of the lac promoter	[41]
pRSet-B-*mCherry*	Used as template for PCR amplification of *mCherry*	Roger Tsien, (University of California, USA)
pCW*-mcherry-parB*	Encodes mCherry-ParB under control of the lac promoter. Derived from pALA2705	This study
pGBKD3-*parS*	Harbors *parS-*frt-cat-frt site for insertion of the *parS* site	[38]

a http://people.ucalgary.ca/~kesander/.

Phages were propagated on *S.* Typhimurium LT2 as plaques in LB soft-agar or as lysates in LB broth as described previously [28]. Phage stocks were filter sterilized with 0.2 µm filters (Fisher Scientific, Aalst, Belgium) and chloroform was added to maintain sterility. Generalized transduction was performed with phage P22 *HT105/1 int-*201 as described previously [28], [29]. This mutant is unable to integrate into the host chromosome as a prophage due to the lack of integrase (Int) activity. To discriminate phage infected from uninfected colonies, plates containing green indicator (GI; [28]) agar were used to indicate cell lysis. The latter medium contains glucose as a carbon source, and a pH indicator dye that turns dark green at sites where phage infection causes cell lysis and the concomitant release of organic acids.

Please note that for clear visualization of spotted or (cross-)streaked bacterial and/or phage populations, agar plates were printed to Whatman filter papers (GE Healthcare, Diegem, Belgium) before photographing.

### β-galactosidase assay

Expression of β-galactosidase (LacZ) was inferred from the hydrolysis of either 5-bromo-4-chloro-3-indolyl-β-galactopyranoside (X-Gal) or o-nitrophenyl-β-D-galactoside (ONPG). X-Gal was typically added to agar plates (40 µg/ml), where its hydrolysis by β-galactosidase yielded an insoluble blue precipitate. For quantitative measurements of *lacZ* expression, Miller units were determined as described previously [30] using the CHCl_3_-sodium dodecyl sulfate permeabilization procedure.

### Construction and screening of a P22 shotgun library

Particles of phage P22 were purified by passing a lysate through a 0.45 µm pore-size filter, after which particles were concentrated by centrifugation (4,000×*g*, 20 min) in the presence of polyethylene glycol (PEG) 8,000 (8%, w/v) and 1 M NaCl. Subsequently, further purification was attained by ultracentrifugation (140,000×*g*, 3 hours) using a layered CsCl step gradient of 1.33, 1.45, 1.50 and 1.70 g/ml. This resulted in a distinct blue band containing the concentrated P22 particles. This band was subsequently collected and dialysed against phage buffer (10 mM Tris-HCl pH 7, 10 mM MgSO_4_, 150 mM NaCl) three times using a Slide-A-Lyzer dialysis cassette (Pierce, Rockford, IL, USA). For DNA extraction, the purified and dialysed phage particles were incubated at 56°C for 1 h in the presence of 0.5% SDS (w/v), 20 mM EDTA and 2 µg/ml proteinase K. Subsequently, DNA was extracted and purified from this mixture by phenol/chloroform [27] and precipitated with Na-acetate/ethanol. Finally, the sample was treated with RNase A (0.1 mg/ml) (Fermentas, St. Leon-Rot, Germany) for 1 h at room temperature to remove any residual RNA. Next, the resulting purified P22 genomic DNA was partially digested with the blunt 4 bp-cutter BsuRI restriction enzyme (Fermentas) and separated by agarose gel electophoresis (1% agarose), after which fragments between 1–2 kb were isolated from the gel using the GeneJET Gel Extraction Kit (Fermentas).

Parallel to this, pFPV-P*_BAD_*–*gfp* (pAA100; [31]) was digested with XbaI and HindIII (Fermentas) to remove *gfp*, and treated with calf intestinal alkaline phosphatase (Fermentas) to prevent self-ligation. The genomic P22 DNA fragments and the cut pFPV-P*_BAD_* vector were subsequently ligated after blunting with T4 ligase and Klenow polymerase (Fermentas), and transformed by electroporation into LT2K7. After plating on LB Ap^100^, this random P22 shotgun library was replica-plated on LB Ap^100^ X-Gal with and without 0.02% arabinose to screen for plasmids able to trigger LacZ expression in LT2K7.

### Whole-genome alignment

Phages containing homologous regions to the region surrounding *pid* were selected with nucleotide Blast [32]. Whole genome alignment was performed manually and was based on the Blast-search results. The resulting conclusions were later confirmed by a progressive Mauve alignment [33] on the full genomes using default settings.

### Construction of bacterial and phage mutants

Strain LT2K7 stems from a random Mu*d*K library, generated as described previously [34], and harbors a translational *lacZ* fusion to the LT2 *dgoT* gene (i.e. *dgoT*::Mu*d*K). In strain LT2 Δ*dgoR*, the *dgoR* gene was deleted via recombineering [35], using an amplicon (Phusion DNA polymerase; Fermentas) prepared on pKD3 [35] with the primers dgoR_pkd3_Fw and dgoR_pkd3_Rev (Table 2). The *cat* cassette replacing *dgoR* was flipped out using pCP20-borne Flp to recombine the two *frt*-sites [36], resulting in a small *frt-*scar followed by a new ribosome binding site [35]. Strain LT2K7 Δ*dgoR* was subsequently constructed by transducing *dgoT*::Mu*d*K to LT2 Δ*dgoR*.

**Table 2:** Primers used throughout the study.

Primer name	Sequence (5′-3′)^a^
dgoR_pkd3_Fw	CAGCTATCGCGGTAAAGTAAGAGAGTTCACATCGAGCACAAGGACTCTCT **GTGTAGGCTGGAGCTGCTTC**
dgoR_pkd3_Rev	CCAGGCGCGCAGATTGGTCGATCCCCAGTCAATTGCGATGTAGCGAGCTGT **CATATGAATATCCTCCTTAG**
pid_YFP_Cm_Fw	CGGTGAGTGCCACTTTTCAACCACAAGAAACTTTATGCAAAGGAGTGGAC **ATGGCTAGCAAAGGAGAAG**
pid_YFP_Cm_Rev	GGGGGGGATAAAAAAGCCGCTTACTTAGCGGCTTGACGTTTGAAGAATG **AGATATCCTCCTTAGTTCCTA**
P22_Int_Fw	AAGGTCGTAGGTTCGACTCCTATTATCGGCACCAGTTAAATCAAATACTTA **AGGAACACTTAACGGCTGACAT**
P22_Int_Rev	GCATACTGTCCAGGTGAGCGCGGGTGATGACATAACAGAGGAACTGAAATG **GTGTAGGCTGGAGCTGCTTC**
P22_parS_Fw	GAATATTTAACATAAAATAAAAATGGGTGTTTACACCCATTTTTATTACA **GATTGTGTAGGCTGGAGCTGC**
P22_parS_Rev	AAAAACCCAATGGAGAATTAGTTAGATTAACCTTGGCAACACTTTAGATA **GGTCTGCTATGTGGTGCTATCT**
pid_Strep_Fw	AACTGAATGAGAAGGTTGCAGCCCTACTGGGCACGCAACAA TGGAGCCACCCGCAGTTCGAAAAA TAA **GTGTAGGCTGGAGCTGCTTC**
pid_Strep_Rev	AAAGCCGCTTACTTAGCGGCTTGACGTTTGAAGAATGA **AGGAACACTTAACGGCTGACAT**
Oligo_C2_Stop	ATGAATACACAATTGATGGGTGAGCGTATTCGC TAATAA GCTCGAAGAAAAAAACTCAAGATTAGACAAG
pid_Fw	GTCA*TCTAGA* **GCCCAAATCGCCGCTTGC**
pid_Rev	CTGA*AAGCTT* **GACATCGGTTATTGCAGAGG**
dgoR_Fw	TCAG*TCTAGA* **TCCGTCGGTCAAAGAGGTGG**
dgoR_Rev	TCAG*TCTAGA* **TTATGCGATGTAGCGAGCTGTC**
pALA_Out_Left	TGAG*GAATTC* **TCTGTTTCCTGTGTGAAATTG**
pALA_Out_Right	TGAG*GAGCTC* **ATGGTCGAGCAGGTATTCAAG**
Mcherry_Fw	TGAG*GAATTC* **ATGGTGAGCAAGGGCGAGG**
Mcherry_Rev	TGAG*GAGCTC* **CTTGTACAGCTCGTCCATGC**
pid_FS_Fw	GAGGTACTCATGACAG
pid_FS_Rev	TCGATTTAAACGCCAC

a When relevant, primer attachment sites are indicated in bold. Relevant restriction enzyme sites are shown in italic. Recombination regions in regular font and the *strep*-tag and two stop-codons introduced in *c2* are underlined.

For the construction of P22 Δ*pid*::*yfp*, the *yfp*-*frt*-*cat*-*frt* cassette was PCR amplified (Phusion DNA polumerase; Fermentas) from plasmid pAc [37] with primers pid_YFP_Cm_Fw and pid_YFP_Cm_Rev (Table 2), and used to replace the *pid* gene in LT2 lysogenized with wild-type P22 via recombineering [35]. Subsequently, the *cat* cassette was flipped out using pCP20-borne Flp to recombine the two *frt*-sites [36], and the resulting P22 Δ*pid*::*yfp* phage was isolated and purified from the corresponding lysogen.

For the construction of P22 Δ*int* Δ*pid*::*yfp*, the integrase gene (*int*) in LT2 lysogenized with P22 Δ*pid*::*yfp* was deleted by recombineering, using a PCR amplicon prepared on pKD3 with primers P22_Int_Fw and P22_Int_Rev (Table 2) [35]. Please note that the *frt*-flanked *cat* cassette was not removed by site specific Flp recombination, since this would interfere with the *frt-*scar already present in the *pid* locus. The resulting P22 Δ*int* Δ*pid*::*yfp* phage could be released by amplifying rare excision events through growth on wild-type LT2 in order to allow detection and purification of plaques. Please note that these phages produced normal turbid plaques and were unable to from true lysogens on LT2.

For the construction of P22 Δ*pid*::*yfp parS*, the *parS-frt-cat-frt* cassette was PCR amplified from pGBKD3-*parS* [38] with primers P22_parS_Fw and P22_parS_Rev (Table 2), and inserted between the *gtrC* and *9* genes in LT2 lysogenized with P22 Δ*pid*::*yfp* via recombineering [35]. Please note that the *frt*-flanked *cat* cassette was not removed by site specific Flp recombination, since this would interfere with the *frt-*scar already present in the *pid* locus.

For the construction of P22 *pid-strep*, a strep-tag encoding sequence (*strep*) was added to the 3′ end of the *pid* open reading frame by recombineering a *strep*-*frt*-*cat*-*frt* amplicon prepared on pKD3 [35] with primers pid_Strep_Fw and pid_Strep_Rev (Table 2) in LT2 lysogenized with wild-type P22. Subsequently, the *cat* cassette was flipped out using pCP20-borne Flp to recombine the two *frt*-sites [36], and the resulting P22 *pid-strep* phage was isolated and purified from the corresponding lysogen. Please note that the C-terminal addition of the strep-tag to Pid was shown to have no effect on the ability of Pid to trigger the *dgo*-operon.

Finally, clear mutants P22 *c2* Δ*pid*::*yfp* and P22 *c2 pid-strep* were constructed by oligo-mediated mutagenesis [39] of the corresponding P22 Δ*pid*::*yfp* and P22 *pid-strep* lysogens in LT2, using olignucleotide Oligo_C2_Stop (Table 2). This oligo introduced two flanking stop-codons after the first 11 amino acids of the P22 C2 repressor. After recombination, transformants were inoculated in LB with wild type LT2 and grown overnight at 37°C to amplify the corresponding clear mutants. Afterwards, P22 *c2* Δ*pid*::*yfp* and P22 *c2 pid-strep* were isolated by plaquing on LT2, and the *c2* mutation was verified by sequencing.

### Construction of plasmids

Plasmid pFPV-P*_BAD_-pid* was constructed by digesting pFPV-P*_BAD_*–*gfp* with XbaI and HindIII (Fermentas), and subsequently replacing *gfp* with *pid*. The latter amplicon (Phusion DNA polymerase; Fermentas) was obtained using primers pid_Fw and pid_Rev (Table 2), digested with XbaI and HindIII prior to ligation. Plasmid pFPV-*dgoR* expresses the LT2 *dgoR* gene under the control of its own promoter, and was constructed by ligating an XbaI digested PCR amplicon of the LT2 *dgoR* locus, obtained with primers dgoR_Fw and dgoR_Rev, into the XbaI site of pFPV25 [40]. Plasmid pCW-*mCherry*-*parB* was constructed by first making an amplicon of the pALA2705 vector [41] with primers pALA_Out_Left and pALA_Out_Right. These primers amplify the entire plasmid except its *gfp* gene, and added an EcoRI and a SacI restriction site at the end of the amplicon. Subsequently, the *mCherry* gene was amplified from pRSet-B-*mCherry* (kind gift from Roger Tsien, University of California, USA) with primers Mcherry_Fw and Mcherry_Rev (Table 2), and both amplicons were digested with EcoRI and SacI prior to being ligated to each other. The resulting plasmid, pCW-*mCherry*-*parB*, expresses an N-terminal fusion of mCherry to ParB under control of an IPTG inducible promoter. Please note, however, that leaky expression of the latter promoter in the absence of IPTG was already sufficient, as mentioned previously [42].

### Site-directed mutagenesis

For site-directed mutagenesis, the “Phusion Site-Directed Mutagenesis Kit” protocol (Thermo Scientific, Epsom, United Kingdom) was followed. As such, plasmid pFPV-P*_BAD_-pid* was used as a template for amplification with primers pid_FS_Fw and pid_FS_Rev (Table 2) for constructing a frame shift mutation in the actual *pid* start codon. After phosphorylating the 5′ ends of the primers according to the manufacturer's instruction, the primer pair was used to PCR amplify pFPV-P*_BAD_-pid* (Phusion polymerase; Fermentas). The resulting linear fragment was purified from an agarose gel using the GeneJET Gel Extraction Kit (Fermentas), subsequently self ligated, and finally transformed by electroporation to *E. coli* DH5α. The resulting pFPV-P*_BAD_-pid* ^FS^ plasmid from transformants selected on LB Ap^100^ was further confirmed by sequencing, prior to transformation to LT2 and LT2K7.

### Protein identification and Western blotting

Samples were lysed in standard lysis buffer containing 50 µl/ml Bugbuster (Novagen, Darmstadt, Germany). Total protein concentration was assessed by the BCA protein assay kit (Novagen) and SDS-PAGE was performed as described previously by Sambrook and Russel [27]). Finally, gels were stained with coomassie [27] and when necessary, silver staining was employed as previously described [43].

For protein identification, the corresponding protein band was excised and trypsin-digested according to the method described earlier [44]. Subsequently, the digested peptides were identified by LC–ESI MS/MS (Thermo Electron, San Jose, CA) and further analyzed using Mascot (Matrix Sciences, London, UK) against the NCBI database (http://www.ncbi.nlm.nih.gov/).

For western-blotting, equal amounts of proteins were separated with PAGE and transferred to a nitrocellulose membrane (Hybond-C Extra; GE Healthcare) by semi-dry electroblotting for 1 hour at 0.15 A using a Trans-Blot SD Semi-Dry Electrophoretic Transfer Cell (Bio-Rad Laboratories) and transfer buffer (50 mM Tris; 40 mM glycine; 0.075% SDS; 20% Methanol). Strep-tagged Pid was subsequently detected by StrepMAB-Classic, an anti-strep monoclonal antibody conjugated with Horse radish peroxidase (IBA, Göttingen, Germany). Horse radish peroxidase activity was assessed with Pierce ECL Western Blotting Substrate (Thermo Scientific), and detected on photo-sensitive film (Hyperfilm ECL; GE Healthcare). The strep-tagged protein ladder (IBA) was used as a molecular ruler and positive control of the blotting process.

### Fluorescence microscopy

Fluorescence microscopy and time-lapse fluorescence microscopy were performed with a temperature controlled (Okolab Ottaviano, Italy) Ti-Eclipse inverted microscope (Nikon, Champigny-sur-Marne, France) equiped with a TI-CT-E motorised condensor, a YFP filter (Ex 500/24, DM 520, Em 542/27), an mCherry filter (Ex 562/40, Dm 593, Em 641/75), and a CoolSnap HQ2 FireWire CCD-camera. For imaging, cells were placed between LB agar pads and a cover glass, essentially as described previously [45], and incubated at 37°C. Please note that for experiments involving LT2 pCW-*mCherry*-*parB*, cells were grown on agar pads of AB-minimal media supplemented with 0.02% D-glucose, 100 µg/ml Uracil and 100 µg/ml Thiamine, Ap^100^, and incubated at 30°C, as described previously [42]. Images were acquired using NIS-Elements (Nikon) and resulting pictures were further handled with open source software ImageJ (Downloaded from http://rsbweb.nih.gov/ij/).

## Supporting Information

Figure S1 Phage ES18 fails to activate the *dgoT*::Mu*d*K fusion in LT2K7. A plaque of phage ES18 grown on a lawn of LT2K7 fails to display LacZ activity (i.e. blue color) on LB X-Gal agar (left panel), while a similar experiment performed on green indicator agar (GI; right panel) confirms the actual infection of LT2K7.
(TIF)

Figure S2 Expression of P22 *pid* segregates asymmetrically between siblings and does not require integration of P22. Exponential phase cultures of LT2 were infected with P22 Δ*int* Δ*pid*::*yfp* (MOI = 0.1) and chased after 30 minutes with the virulent P22 H5 mutant (MOI = 20) to lyse cells not destined for non-lytic development of P22 Δ*int* Δ*pid*::*yfp*. Images A and B depict clonal microcolonies displaying asymmetrical segregation of *yfp* expression. Phase contrast (left panels) and corresponding YFP epifluorescence (right panels) images are shown, and the time after infection with P22 Δ*int* Δ*pid*::*yfp* is indicated on the frame.
(TIF)
