# Supplementary figures and images for: Expression of a Novel P22 ORFan Gene Reveals the Phage Carrier State in Salmonella Typhimurium
Source: PLoS Genet. 2013 Feb 14;9(2):e1003269. doi: 10.1371/journal.pgen.1003269 (PMC3573128; doi:10.1371/journal.pgen.1003269)

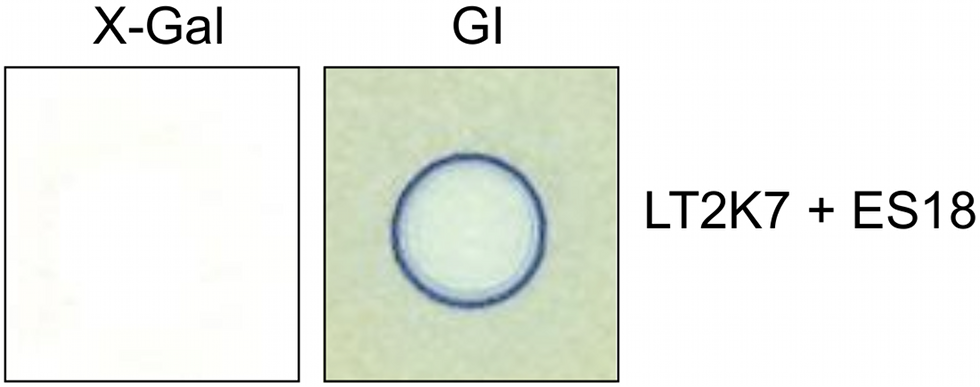

Supplement: Figure S1 — Phage ES18 fails to activate the dgoT::MudK fusion in LT2K7. A plaque of phage ES18 grown on a lawn of LT2K7 fails to display LacZ activity (i.e. blue color) on LB X-Gal agar (left panel), while a similar experiment performed on green indicator agar (GI; right panel) confirms the actual infection of LT2K7. (TIF) [file pgen.1003269.s001.tif]

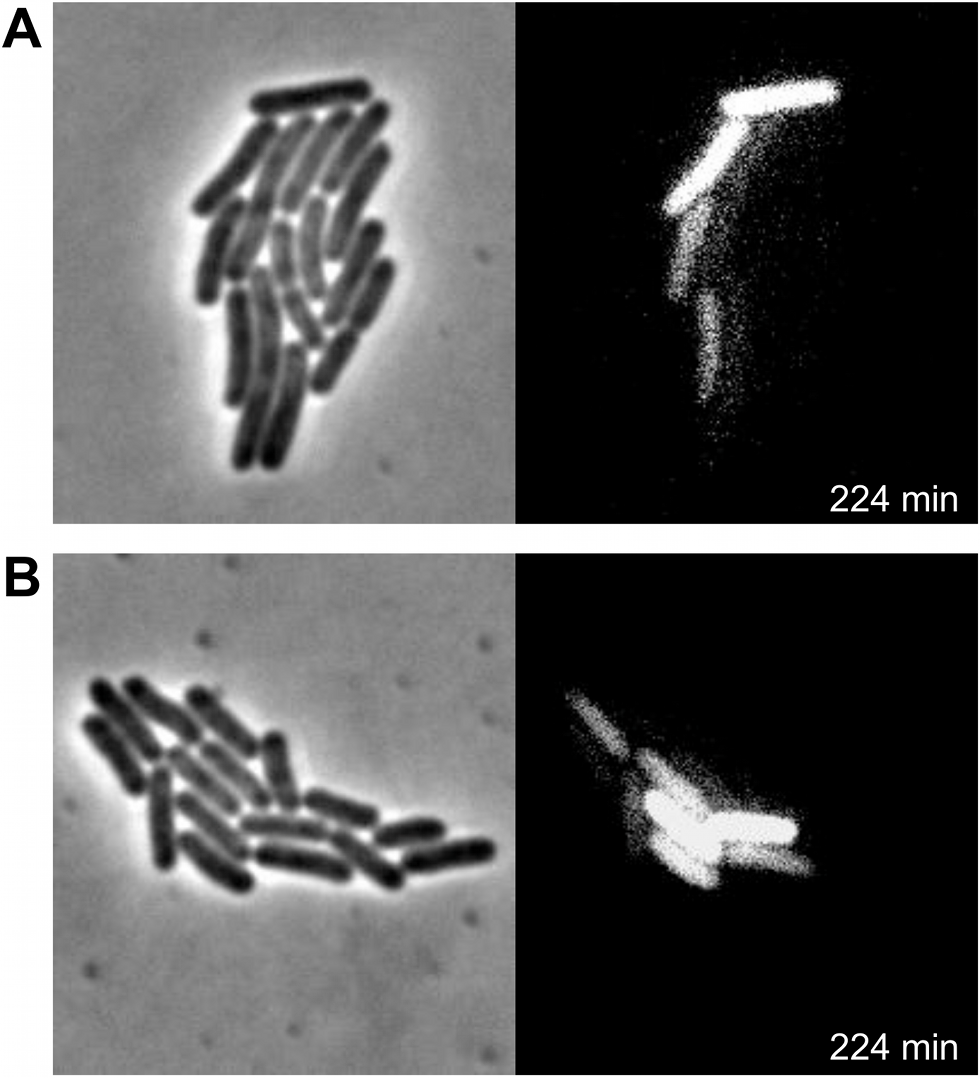

Supplement: Figure S2 — Expression of P22 pid segregates asymmetrically between siblings and does not require integration of P22. Exponential phase cultures of LT2 were infected with P22 Δint Δpid::yfp (MOI = 0.1) and chased after 30 minutes with the virulent P22 H5 mutant (MOI = 20) to lyse cells not destined for non-lytic development of P22 Δint Δpid::yfp. Images A and B depict clonal microcolonies displaying asymmetrical segregation of yfp expression. Phase contrast (left panels) and corresponding YFP epifluorescence (right panels) images are shown, and the time after infection with P22 Δint Δpid::yfp is indicated on the frame. (TIF) [file pgen.1003269.s002.tif]
